# Insights into the Role of GILT in HLA Class II Antigen Processing and Presentation by Melanoma

**DOI:** 10.1155/2009/142959

**Published:** 2009-11-24

**Authors:** Duncan L. Norton, Azizul Haque

**Affiliations:** Department of Microbiology and Immunology, Charles Darby Children's Research Institute and Hollings Cancer Center, Medical University of South Carolina, 173 Ashley Avenue, Charleston, SC 29425, USA

## Abstract

Metastatic melanoma is one of the deadliest of skin cancers and is increasing in incidence. Since current treatment regimens are ineffective at controlling and/or curing the disease, novel approaches, such as immunotherapy, for treating this malignant disease are being explored. In this review, we discuss potential melanoma antigens (Ags) and their role in utilizing the HLA class II pathway to elicit tumor Ag-specific CD4+ T cell responses in order to effectively induce long-lasting CD8+ antitumor memory. We also discuss the role of endolysosomal cathepsins and Gamma-Interferon-inducible Lysosomal Thiol reductase (GILT) in Ag processing and presentation, and at enhancing CD4+ T cell recognition of melanoma cells. This review also summarizes our current knowledge on GILT and highlights a novel mechanism of GILT-mediated immune responses against melanoma cells. At the end, we propose a strategy employing GILT in the development of a potential whole cell vaccine for combating metastatic melanoma.

## 1. Introduction

Rising incidences of melanoma make it one of the rapidly growing cancers plaguing western populations. It is the 8th leading cause of cancer deaths in the US with an estimated 68,720 new cases diagnosed in 2009 [1–5]. Current melanoma therapies such as surgery, radiation, and chemotherapy are effective against early stage localized tumors [1–4]. However, these therapies fail to treat and cure large malignant tumors which generally prove to be fatal. These therapies are also extremely damaging and toxic to the patient, suggesting a need for the development of new therapies. Immunotherapy is an extremely attractive therapy against melanoma as it is associated with a high cure rate and could have little to no potential side effects while providing long term protection against a recurrence of the disease. Long-lasting immune responses and protection against melanoma is largely dependent upon the activation of helper CD4+ and cytotoxic CD8+ T cells [6, 7]. Current studies suggest that malignant melanomas can constitutively express Human Leukocyte Antigen (HLA) class I and II molecules which are essential for the stimulation of CD8+ and CD4+ T cells [8, 9]. Also, strong CD8+ and CD4+ T cell immune memory responses to the presentation of tumor antigens (Ags) seem to require processing and crosspresentation by professional antigen presenting cells (APCs) [10, 11]. A key factor that distinguishes APCs such as dendritic cells (DC), macrophages, and B cells from melanoma cells is the fact that APCs express an abundance of adhesion and costimulatory molecules (e.g., CD80 and CD86) that allow for prolonged interaction between HLA complexes and T cells [12]. In the case of the HLA class II pathway, Ag processing and presentation by APCs rely heavily on proteases in intracellular endosomes and lysosomes which give rise to a large array of peptides for display to CD4+ T cells.

Inside APCs, reactions crucial to the processing of Ags and large peptides such as proteolysis and the reduction of disulfide bonds are highly effective; ultimately giving rise to HLA class II-peptide complexes available for presentation to T cells. A potential problem that afflicts HLA class II presentation is the spontaneous cysteinylation of peptides and Ags both in vitro and in vivo [11, 13]. This cysteinylation is due to interaction with cystine within biological fluids [10, 11]. We have previously shown that melanoma cells expressing HLA class II molecules fail to effectively process oxidized or cysteinylated peptides [11]. This disruption in processing of peptides ultimately ends with a modified epitope presentation to CD4+ T cells and a lack of an immune response. We have also shown that the enzyme Gamma-IFN-inducible Lysosomal Thiol reductase (GILT) is highly expressed in professional APCs, but absent or slightly expressed in human melanomas [11]. The expression of GILT has been found to restore the processing of cysteinylated melanoma tumor Ags and CD4+ T cell recognition of tumors cells [11]. A lack of GILT in melanoma cells has been found to alter the processing of both endogenous and exogenous proteins/peptides within the tumor cells [14]. The absence of GILT within melanomas also allows for a differential display of antigenic peptides which results in an escape from the class II pathway of immune recognition. The reduction of disulfides is important for the processing of tumor Ags such as tyrosinase, gp-100, Mart-1, and NY-ESO-1, which all contain a large number of cystiene residues [15–18]. Thus, the expression of GILT is needed in melanoma cells and APCs for reductive processing and presentation of these melanoma Ags to T cells.

Professional APCs express high levels of HLA class II molecules and GILT which are crucial for Ag processing and presentation to CD4+ T cells. While many studies have focused on increasing the immunogenicity of melanoma cells, one of the most efficient methods is to have the tumor cells present their own Ags and peptides [10, 11, 19, 20]. We and others have shown that melanoma tumors do indeed have HLA class II molecules, but these molecules are often expressed at low levels [10, 11, 21, 22]. Thus, this review will focus on GILT; its function in improving Ag processing and presentation within melanoma cells and we will discuss the implications it may have in the treatment of patients with metastatic melanoma.

## 2. Potential Self Ags for Use in Melanoma Immunotherapy

Melanoma cells express a wide variety of self proteins that have antigenic properties and could induce potent immunogenic responses against the tumor. The most widely known and expressed melanoma Ags are MART-1, NY-ESO-1, gp100, and tyrosinase. These Ags have been shown to elicit both cytotoxic CD8+ and helper CD4+ T cell responses. Each of these Ags has been extensively studied, and potential immunodominant epitopes have been identified for the development of vaccine reagents against metastatic melanoma. In this section, we will discuss these self Ags and their antigenic determinants that may elicit both CD4+ and CD8+ T cell responses against HLA class I and II expressing melanoma cells.

MART-1, also known as Melan-A, is a commonly detected melanoma-associated Ag. Since its discovery in 1994, MART-1 has been the focus of the development of strategies to target melanoma via the immune system [23, 24]. A number of naturally occurring HLA class I and II epitopes have been identified from MART-1 and have since become the basis of many clinical trials. One such peptide is the class I-restricted MART-1_27–35_ (AAGIGILTV) which is capable of eliciting an antitumor CD8+ T cell response [24]. Two slightly longer versions of this peptide: Melan-A_27–40_ (AAGIGILTVILGVL) and Melan-A_25–36_ (EEAAGIGILTVI), have been shown to elicit CD4+ T cell responses [22]. These particular peptides are extremely attractive targets for inducing antimelanoma immunity as they can be further processed into the immunogenic class I-restricted peptide which is capable of eliciting cytotoxic T cell (CTL) responses. Similarly, the HLA-DR4-restricted peptide MART-1_51–73_ (RNGYRALMDKSLHVGTQCALTRR) has been shown to induce high CD4+ T cell responses in conjunction with the class I peptide MART-1_27–35_ within melanoma patients [25]. The ability of each of these peptides to generate effective CD4+ and CD8+ antitumor immune responses makes them attractive targets for treating melanoma. 

NY-ESO-1 is a tumor-associated Ag that is found in a multitude of cancers such as melanoma, breast, prostate, and lung [26]. Over the past decade, NY-ESO-1 has become one of the many targets for potential immunotherapy against melanoma. Within melanoma cells, a number of HLA class I and HLA class II-restricted NY-ESO-1 epitopes have been identified as immunodominant epitopes. NY-ESO-1_60–72_ (APRGPHGGAASGL) is a relatively long, naturally occurring peptide that is restricted to HLA-B7 molecules [27], which is commonly expressed in all populations regardless of geographic location [28, 29]. The HLA class II-restricted peptide NY-ESO-1_116–135_ (LPVPGVLLKEFTVSGNILTI) has also been shown to be capable of stimulating cytokine production by CD4+ T cells [18]. It is interesting to note that NY-ESO-1_116–135_ peptide-specific T cells are also able to recognize B-lymphoblastoid cells pulsed with the whole protein [18]. This suggests that NY-ESO-1 can be naturally processed by tumors as well as professional APCs, and then presented via the class II pathway for the induction of Ag-specific antitumor immunity. These two peptides: NY-ESO-1_60–72_ and NY-ESO-1_116–135_, could be combined in therapy to cause regression of melanoma as well as other tumors.

 Gp100, also known as pmel17, is a melanosomal matrix protein involved in the synthesis of melanin. It was originally identified as a potential tumor Ag after tumor infiltrating lymphocytes were seen in cancer regressing melanoma patients [30]. Gp100 has since been studied for its potential uses in different immunotherapies for tumor regression and establishing lasting antitumor memory. Many Gp100 epitopes have been predicted for prospective use in the treatment of melanoma, one of which is the gp100_44–59_ (WNRQLYPEWTEAQRLD) epitope. Gp100_44–59_ has been previously shown to stimulate CD4+ T cells [31], and has also been reported as a natural epitope [32]. Another potential peptide is the HLA-A2 restricted gp100_154–162_ (KTWGQYWQV) epitope which is capable of eliciting CD8+ T cell responses when presented by mature DC [33]. Since both APCs and tumors naturally process and present these tumor-associated peptides to both CD8+ and CD4+ T cells, they could be used in devising new immunotherapeutics against metastatic melanoma. 

Tyrosinase is a type 1 transmembrane protein that is often found in melanoma. Tyrosinase has previously been studied and has been found to encode multiple epitopes that are recognized by CD4+ T cells. Of these multiple epitopes, tyrosinase_56–70_ (QNILLSNAPLGPQFP) has been suggested as a possible immunodominant epitope and shown to elicit CD4+ T cell responses [33]. Another tyrosinase peptide is the HLA-A restricted tyrosinase_243–251_ (KCDICTDEY) which is unique in the sense that it contains two cysteine residues, since many HLA class I epitopes identified have few to no cysteine residues [34]. Tyrosinase_243–251_ is naturally processed and is recognized by CTLs [34], making it an attractive target in conjunction with tyrosinase_56–70_ for developing treatments against malignant melanoma.

MART-1, NY-ESO-1, gp100, and tyrosinase contain multiple cysteine residues which are susceptible to oxidation reaction. GILT expression in melanoma cells may help process these Ags, or their cysteinylated peptides, to functional epitopes for delivery to T cells via HLA molecules. Immunodominant epitopes from Ags expressed by melanoma tumors could be the focus of therapeutics to aid in the treatment of melanoma patients. One potential treatment option could be the development of a variety of vaccines to challenge the patient's immune system. Possible strategies may include whole cell vaccines, various dendritic cell vaccines, and peptide vaccines where HLA class I and II-restricted immunodominant epitopes from multiple tumor Ags are used. These vaccine materials are few examples that could stimulate the immune system via the HLA class I and II pathways leading to tumor regression and induction of lasting antitumor immune memory.

## 3. Importance of HLA Class II Pathway in Direct Ag Presentation by Melanoma Cells

HLA class II molecules can bind both endogenously and exogenously derived antigenic peptides and stimulate CD4+ T cells [35]. In order for CD4+ T cells to recognize these Ags/peptides, they must first be processed by APCs or tumor cells, loaded onto the HLA class II dimer, and transported to the cell surface. Professional APCs express high levels of HLA class II proteins and are efficient in delivering processed peptides to CD4+ T cells [36]. HLA class II *α β* heterodimers are synthesized within the endoplasmic reticulum (ER) of APCs and form a complex with the glycoprotein invariant chain (Ii) which prevents premature peptide loading into class II molecules [37–39]. Ii also facilitates the transport of HLA class II *α β* heterodimers to the endolysosomal compartments via the trans-Golgi network [40]. Once inside the endolysosomal compartment, acidic proteases, such as cathespins S and L, begin to degrade Ii into smaller intermediate peptides with the end result being the class II-associated Ii peptide (CLIP) [41–44]. CLIP blocks the peptide binding groove until it is removed, effectively preventing premature peptide loading. Processing of Ags also occurs during this time by other acidic proteases such as cathepsins D and B [41, 45, 46]. These acidic proteases unfold, cut, and splice both endogenous and exogenous Ags into smaller peptides. The nonclassical molecule, HLA-DM, has a unique role within the class II pathway [47, 48]. HLA-DM chaperones the removal of CLIP from the peptide binding grove and affects the peptide repertoire by influencing high affinity peptides to bind to the peptide binding pouch, effectively forming a HLA class II-peptide complex [35, 47, 48]. The HLA class II-peptide complex is then transported to the cell surface for presentation to CD4+ T cells. Upon TcR interaction with the HLA class II-peptide complex, members of the B7 family (CD80/CD86) interact with CD28 on the T cell surface. This interaction lowers the signaling threshold needed for stimulation and cytokine production [49, 50]. Upon stimulation, CD4+ T cells can help direct an immune response against the targeted Ag(s). Since melanoma cells express their own tumor Ags that contain class II-restricted epitopes, activation of the HLA class II pathway could induce an effective antitumor immune response against the malignant disease.

 Current animal model studies indicate that established small tumors can be reduced by actively immunizing the host against tumor cell Ags. The immunization of animals with specific tumor cells transduced to express HLA class II and the costimulatory molecules CD80/CD86 have been shown to halt tumor progression and establish an immune memory against the specific tumor [12]. The antitumor response elicited by the vaccines is dependent on CD4+ T cell activation through HLA class II molecules on both APCs and tumor cells. Since melanoma cells express HLA class II molecules, they can act as APCs and present their own tumor Ags to CD4+ T cells. CD4+ T cells, which recognize these tumor epitopes, could be activated and would then be able to recruit professional APCs as well as CTLs and NK T cells. Thus, by inducing melanoma tumors to present their own Ags, the host would be able to naturally reduce the tumor without the side effects of current therapies.

## 4. Acidic Proteases Such As Cysteinyl and Aspartyl Cathepsins Are Important for Ag Processing and HLA Class II Presentation by Melanoma Cells

**Figure 1:**
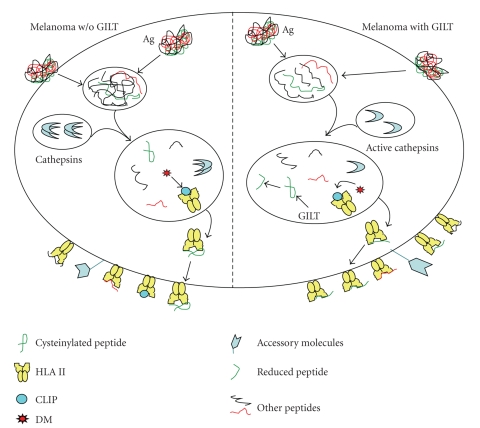
Norton and Haque.

Ag processing and presentation are important for successful CD4+ T cell stimulation. Intracellular proteases such as cysteinyl and aspartyl cathepsins (Cat S, B, D,) are essential for degradation of endogenous and exogenous Ags, and each performs an important role in the processing of peptides for presentation (Figure 1). Cathepsin B can also aid in Ag presentation by upregulating class II protein expression in APCs [41, 46]. Cathepsin D has a very unique role in Ag presentation by degrading and splicing Ags into short peptides [45, 46]. Peptides cannot be loaded into the class II binding grove until CLIP is removed by the nonclassical molecule, HLA-DM [48]. After HLA-DM has successfully chaperoned the insertion of a high-affinity peptide, stable HLA class II-peptide complexes can then be presented on the cell surface for CD4+ T cell recognition [51]. 

We have previously shown that the active forms of cysteinyl and aspartyl cathepsins are upregulated in melanoma cells transfected with GILT [46]. Our laboratory has also demonstrated that GILT colocalizes with Cat D and B, further suggesting that GILT is crucial for Ag processing and presentation via the class II pathway [46]. This also suggests that melanoma cells expressing GILT may be able to process more tumor Ags and produce functional epitopes for class II presentation. Our current studies suggest that the protease activities of cathepsin S, B, and D are increased in GILT-expressing melanoma cells as compared to melanoma cells lacking GILT (unpublished data). This upregulation of acidic proteases raises the possibility that more functional epitopes could be generated for HLA class II presentation by melanoma cells. Since melanoma cells express many self proteins that are antigenic, introduction of GILT in these cells could be beneficial for generating a pool of functional tumor peptides. This would then lead to CD4+ T cell recognition and ultimately tumor destruction by immune effector cells such as CTL and NK T cells.

## 5. The Insertion of GILT into Melanoma Cells Restores CD4+ T Cell Recognition

GILT is a lysosomal reductase, abundantly expressed in professional APCs, but absent or undetectable in melanoma cells and other tumors [52]. However, GILT can be induced in melanoma and other tumor cells when treated with IFN*γ* [53]. Within APCs, GILT is found in its proform in early endosomes, while the mature form is found in multivesicular late endosomes and multilaminar lysosomes [54]. GILT is active at low pH and catalyzes the breaking of disulfide bonds, making Ags/peptides susceptible to degradation by acidic cathepsins [55, (Figure 1)]. Experimental evidence suggests that GILT is required for processing of posttranslationally modified/oxidized epitopes for HLA class II-mediated presentation [56]. We have previously reported that GILT is regulated by STAT-1 instead of class II transactivator (CIITA) which is important for the activation of HLA class II gene loci [57].

**Figure 2:**
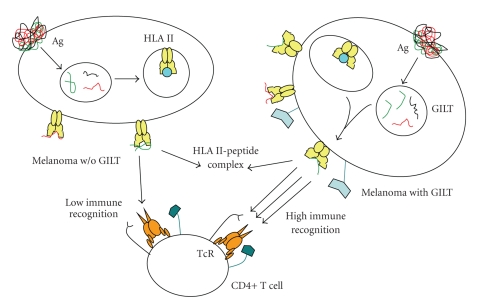
Norton and Haque.

 GILT expression has been shown to enhance Ag processing and CD4+ T cell recognition in vitro and in vivo [11, 46, 56]. An alteration in the expression of tumor Ags or disturbance in the HLA presentation pathway may be the reason tumor cells evade immunogenic ablation [58]. Thus, the restoration of the HLA class II peptide presentation pathway in melanoma may enhance the ability of the tumor cells to promote immunological destruction and surveillance. However, T cell responses to tumor cells are often low due to the lack of direct presentation of modified peptides bound to class II molecules (Figure 2). The formation of class II-peptide complexes is also significantly lower in tumors when compared against professional APCs [25]. Therefore, the lack of GILT in melanoma cells might partially explain why HLA class II-peptide complexes play such a limited role in stimulating T cell responses against tumors. We have demonstrated that the insertion of GILT in melanoma cells in vitro has the ability to restore CD4+ T cell recognition [11]. Other studies have shown that CD4+ T cells specific for select Ags were reduced in animals lacking the novel enzyme GILT [56]. Collectively, GILT expression can play an important role in enhancing CD4+ T cell recognition of melanoma cells (Figure 2). However, a complete understanding of GILT's role within the HLA class II pathway has yet to be determined; perhaps may contribute to the regulation of other factors such as costimulatory or accessory molecules and intracellular proteases.

## 6. Implications of GILT in Devising a Whole Cell Vaccine against Melanoma

We have previously shown that GILT upregulates Ag processing and presentation via the HLA class II pathway [10, 46], and the regulation of GILT protein expression is dependent on STAT-1 while independent of CIITA [57]. Our unpublished data suggest that GILT-expressing melanoma cells generate more functional epitopes when compared to non-GILT expressing melanoma. This greater generation of epitopes could allow for not only improved immune recognition but also the spreading of epitopes to professional APCs and non-GILT expressing tumor cells, as well as bystander cells. These professional and nonprofessional APCs as well as tumors such as melanoma could then be able to present this repertoire of epitopes to CD4+ T cells, further priming an immune response and leading to the ablation of tumor cells. 

Successful destruction of melanoma cells would also require optimum immune recognition and the development of lasting immune memory. In vivo, melanoma cells express a multitude of tumor-associated Ags which may be targeted by T cells through the HLA class II pathway, ultimately leading to tumor regression. Since these Ags are capable of stimulating the immune system, tumor samples could be taken from patients, have GILT expression restored within these tumor cells, and then given back to patients as a whole cell vaccine. This whole cell vaccine would allow for a greater reduction of disulfide bonds by the melanoma, an important difference from other whole cell vaccines such as Canvaxin [59]. Also, unlike Canvaxin which uses three allogenic melanoma lines that express a large number of tumor Ags [60], our proposed strategy could focus an immune response against patient specific tumor Ags expressed by the patient's own genetically modified tumor. This approach could still take advantage of both cellular and humoral responses while enhancing the antigen processing and presentation ability of the genetically modified melanoma. In this scenario, GILT expression would upregulate key immune components such as Ag processing and presentation molecules and proteases. This upregulation would lead to greater tumor Ag processing and presentation via the HLA II pathway (Figure 2). CD4+ T cells would then recognize GILT-expressing cells and recruit other immune cells and APCs in order to destroy the tumor cells. The death of the GILT expressing melanoma would generate a large pool of Ags and peptides which could then be processed and presented by professional APCs. After successfully stimulating an antitumor immune response against the GILT expressing melanoma, the patient's melanoma cells, not expressing GILT, could be recognized by both immune effector cells, CTLs and NK T cells, and immune regulating CD4+ T cells; further improving tumor regression. Upon tumor regression and remission, the patient could be further protected against any reoccurrence of the melanoma by a potent antitumor memory response, successfully halting future disease formation.

## 7. Conclusions

Metastatic melanoma is an intricate disease that is extremely difficult to treat. Novel therapeutic approaches such as immunotherapy need to be pursued in order to effectively treat patients with metastatic melanoma. Since metastatic melanoma cells express a wide variety of tumor associated Ags, they could be presented to the immune system via the HLA class II pathway. In this review, we have discussed previous studies on the molecular and cellular functions of GILT, and its effect on HLA class II presentation by melanoma tumors. We have also discussed the implications of GILT in the development of whole cell vaccines against metastatic melanoma. Further studies focusing on the effect of GILT in melanoma, both in vivo and in vitro, need to be pursued in order to develop successful therapies for use against metastatic melanoma.

## Abbreviations

HLA: Human leukocyte antigen
GILT: Gamma-Interferon-inducible Lysosomal Thiol reductase
Cat: Cathepsins
TcR: T cell Receptor
CTL: Cytotoxic T cells.
